# Successful treatment of bilateral open calcaneal fractures with concomitant lower extremity injuries: A case report

**DOI:** 10.1186/1757-1626-1-194

**Published:** 2008-09-30

**Authors:** Melih Güven, Namık Kemal Özkan, Murat Çakar, Umut Yavuz, Budak Akman, Barış Kadıoğlu

**Affiliations:** 1 The Hospital of University of Abant Izzet Baysal, Department of Orthopaedics and Traumatology, Bolu, Turkey; 2 Göztepe Training and Research Hospital, 2nd Orthopaedic and Traumatology Clinic, Istanbul, Turkey; 3 Bağcılar Training and Research Hospital, Orthopaedic and Traumatology Clinic, Istanbul, Turkey

## Abstract

Open calcaneal fractures are high morbidity injuries and the risk of complications depends on the concomitant injuries, on the size and the position of the traumatic wound. A 53-year-old male patient with bilateral open calcaneal fractures and associated concomitant lower extremity injuries such as subtalar dislocation, talonavicular dislocation and open distal tibial metaphyseal fracture was immediately operated by percutaneous Kirschner wire fixation combined with external fixators. He was able to walk with full weight bearing without any assistance at the end of the first postoperative year. Early aggressive debridement and irrigation followed by fixation with percutaneous Kirschner wires and external fixator can supply bony alignment in open comminuted calcaneal fractures associated with concomitant lower extremity injuries and should be considered for the healthy and active patients before primary arthrodesis.

## Background

Open calcaneal fractures are potentially problematic, destructive hind foot injuries and can result in a wide range of outcomes regardless of the initial treatment modalities [1,2]. There are many relatively poor outcomes in the literature. The reported complications rate after open calcaneal fractures changes between 7,7% – 37% such as wound infection, osteomyelitis and amputation [3-7]. The risk of complications depends on the concomitant injuries, on the size and the position of the traumatic wound [8]. Unsatisfactory outcomes may result from neurogenic pain, infection, malunion, arthrosis and bony impingement [2]. To our knowledge, there is no previous report in the English language medical literature about the treatment and results of bilateral open calcaneal fractures with talonavicular and subtalar dislocations and open tibial fracture. The purpose of this paper is to report the result of the operative technique that was applied immediately for bilateral open calcaneal fractures with associated concomitant lower extremity injuries.

## Case presentation

**Figure 1:**
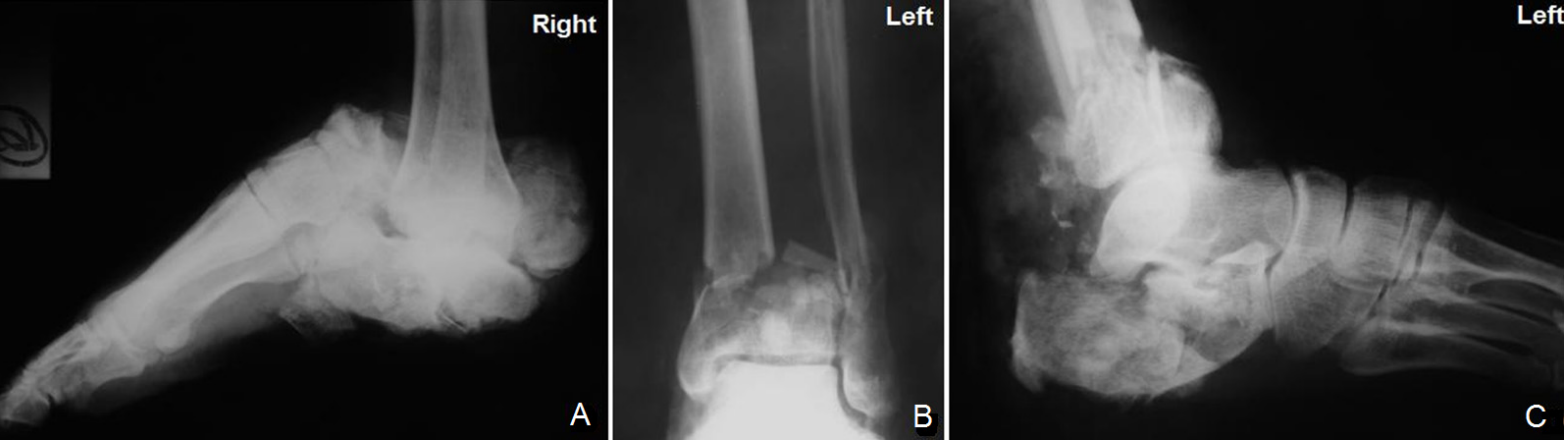
A lateral radiograph of the right foot (A) and anteroposterior (B) and lateral (C) radiographs of the left lower extremity at the time of injury.

A 53-year-old male worker with a history of falling approximately 10 m height on his feet was referred to our hospital. He sustained multiple injuries such as stable burst fracture of third lumbar vertebrae, left anterior shoulder dislocation and bilateral open comminuted calcaneal fractures. Plain radiographs showed also subtalar and talonavicular dislocation on the right foot and open distal comminuted tibial fracture on the left lower extremity (Figure 1).

After hemodynamic stabilization, a through wound evaluation and detailed neurovascular assessment of the lower limbs were made. There was 10 cm long open plantar wound lesion on the right foot and 8 cm long open wound lesion on the medial side of the left foot through the medial malleolus. His neurovascular examination was intact. Immediate debridement and irrigation were performed in the emergency room. A tetanus prophylaxis was administered and intravenous antibiotics, consisting of a first-generation cephalosporin (cefazolin, 1 gr/8 hr), an aminoglycoside (gentamycin 5 mg/kg/day) and an antianaerobic prophylaxis (metranidazole 500 mg/12 hr) were started.

All open fractures on his lower extremities were classified using the Gustilo-Anderson classification [9] as grade 3A. His calcaneal fractures were classified according to the Sanders classification [10] as type 4.

**Figure 2:**
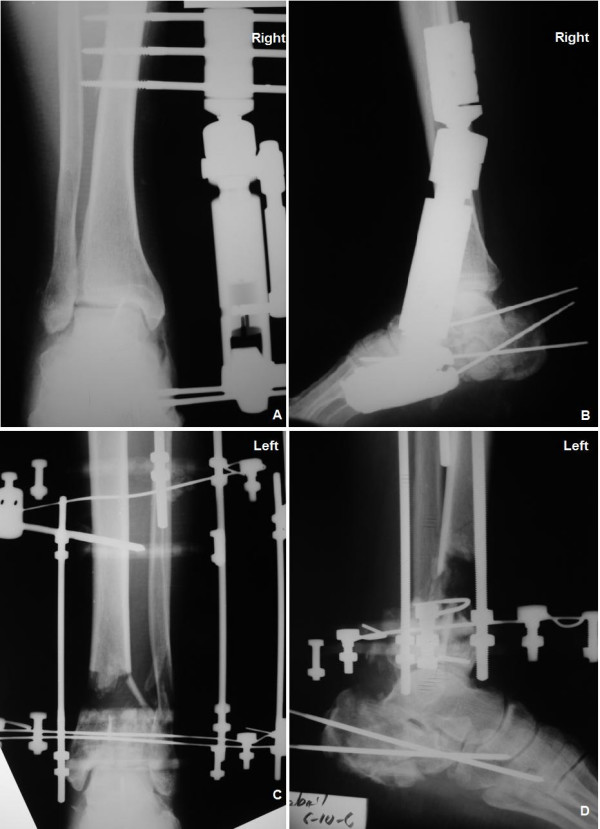
Early postoperative anteroposterior and lateral radiographs of both feet.

He was taken to the operating room in the same day. Under general anesthesia, his left shoulder dislocation was reduced closely. Debridement combined with irrigation with at least 10 liters of normal saline solution for both lower extremities were repeated. Under fluoroscopic control, left distal comminuted tibial fracture was reduced and a pre-constructed Ilizarov fixator that consisted of three 140 mm circular rings was mounted on the tibia. Percutaneous fixation with two 2,5 mm Kirschner wires was applied to the left calcaneal fracture from the calcaneal tuberosity to provide as far as possible the entirety of the posterior articular surface of the calcaneus (Figure 2C and 2D).

A lateral longitudinal incision below the lateral malleolus was made on the right ankle. The talonavicular dislocation was reduced and fixed by a 2.5 mm Kirschner wire from the posterior part of the talus into the navicula. Comminuted calcaneal fracture was reduced as far as possible and stabilized with two percutaneously placed 2.5 mm Kirschner wires. The uniplaner external fixator was applied between the right tibia and first metatarsal to secure the ankle and subtalar joint in neutral position for bony alignment (Figure 2A and 2B). The traumatic wounds were closed primarily.

Intravenous antibiotics were continued 72 hours after the surgery and low molecular weight heparin prophylaxis was administered during the hospital course. He was discharged from the hospital on the tenth postoperative day. There were no complications such as infection and skin necrosis during the follow-up. The patient had four months of non-weight-bearing activity, with active range of motion of the left subtalar and ankle joints.

**Figure 3:**
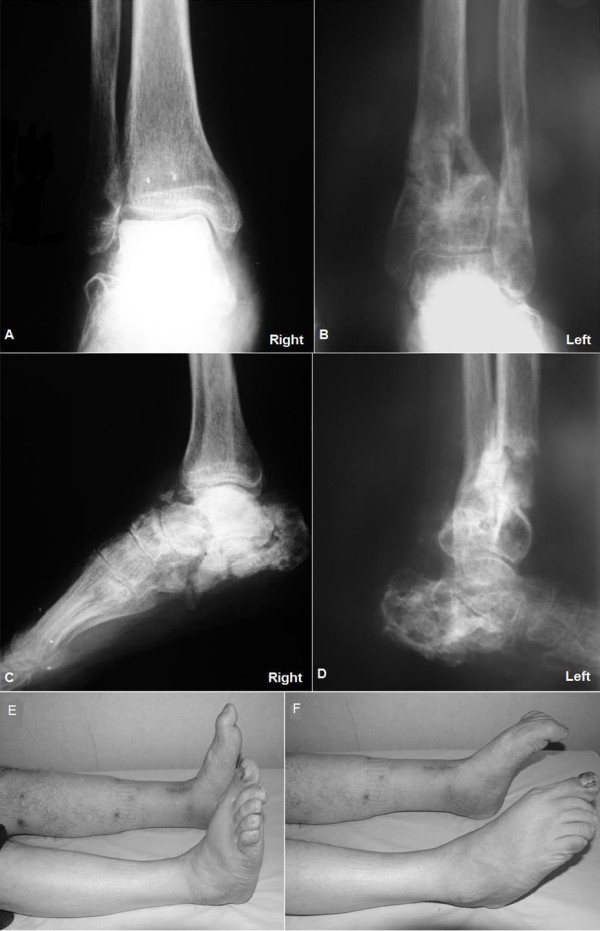
**Radiographs (A-D) and clinical pictures (E, F) at the end of the first postoperative year**.

The uniplaner external fixator on the right lower extremity and the Kirschner wires on the both feet were removed four months after the index surgery due to the radiographic healing of both calcanei. At the sixth month follow-up he was able to walk with full weight bearing on the both lower extremities with assistance of crutches. On the physical examination he had no pain on the both feet with mild pain on the left ankle. Plain radiographs showed left tibial non-union and therefore autogenous corticocancellous bone grafting from iliac crest was applied as a second operation. At the ninth month follow-up plain radiograhs showed complete healing of the tibial nonunion and Ilizarov external fixator was removed (Figure 3A–D).

At the end of the first postoperative year, range of motion of his right and left ankle were, respectively, dorsiflexion 0 and 5 degrees; plantar flexion 35 and 20 degrees (Figure 3E and 3F). His both subtalar ankle motion as inversion and eversion were, respectively, 5 degrees and 0 degrees. The American Orthopaedic Foot and Ankle Society Hind foot score [11] was 65 in the right and 69 in the left foot. He was able to walk independently.

## Discussion

The goals of open calcaneal fracture management include timely healing of the soft tissue without infection and maintenance of bony alignment [2]. However the orthopedic literature remains controversial regarding the optimal management of compound calcaneal fractures. Isolated open calcaneal fractures are relatively rare and appear to be associated with multiple injuries [2]. Some authors recommended that management of the soft-tissue disruption and avoidance of infection should be the initial treatment focus, rather than fracture stabilization [3,7]. On the other hand; the stability provided by early internal fixation of open fractures is believed to diminish the risk of infection and promote healing of the injured soft tissues [12,13]. Heier et al. [6] reported that aggressive irrigation, debridement and fracture stabilization would minimize the rate of soft-tissue infection, calcaneal osteomyelitis and limb amputation. They concluded that patients with deep infection or osteomyelitis were more likely to require an amputation.

Although general treatment recommendation for non-reconstructible fractures of the posterior facet is early or late subtalar arthrodesis [2]; immediate minimally invasive fixation with percutaneous pinning and external fixation should be considered for the healthy and active patients. It is not possible to restore all parts of comminuted calcaneal fracture with Kirschner wires. But the aim of percutaneous fixation in the presented case was to provide the entirety and the bony alignment of the calcaneus. Our patient was able to walk with full weight bearing on the both lower extremities without any assistance at the end of the first postoperative year. However posttraumatic arthritis is possible with this severe injury. That may become a problem in the future, but the percutaneous fixation technique used here does not eliminate the option of arthrodesis.

## Conclusion

Early aggressive debridement and irrigation followed by percutaneous Kirschner wire fixation combined with external fixator can supply bony alignment in open comminuted calcaneal fractures associated with concomitant lower extremity injuries. After all, the patients with open calcaneal fractures should be forewarned that extensive and staged surgical procedures may be necessary.

## Competing interests

The authors declare that they have no competing interests.

## Authors' contributions

MG and NKÖ contributed to manuscript conception and design, carried out the literature research, manuscript preparation and manuscript review. MÇ and UY contributed to manuscript preparation and manuscript review. BA contributed to manuscript conception and design. MG and BK revised the manuscript for important intellectual content. All authors read and approved the final manuscript.

## Consent

Written informed consent was obtained from the patient for publication of the study and any accompanying images. A copy of the written consent is available for review by the Editor-in-Chief of this journal.
